# Cancer Antigen 125 Levels at Time of Ovarian Cancer Diagnosis by Race and Ethnicity

**DOI:** 10.1001/jamanetworkopen.2025.1292

**Published:** 2025-03-20

**Authors:** Anna Jo Bodurtha Smith, Emily Gleason, Sneha Kadiyala, Xingmei Wang, Elizabeth A. Howell, Anne Marie McCarthy

**Affiliations:** 1 Division of Gynecologic Oncology, Department of Obstetrics and Gynecology, University of Pennsylvania Health Systems, Philadelphia; 2 Department of Obstetrics and Gynecology, University of Pennsylvania Health Systems, Philadelphia; 3 Leonard Davis Institute of Health Economics, University of Pennsylvania Health Systems, Philadelphia; 4 Penn Center for Cancer Care Innovation, Abramson Cancer Center, University of Pennsylvania Health Systems, Philadelphia; 5 Perelman School of Medicine, University of Pennsylvania, Philadelphia; 6 University of Pennsylvania, Philadelphia; 7 Department of Biostatistics, Epidemiology and Informatics, Perelman School of Medicine, University of Pennsylvania, Philadelphia

## Abstract

**Importance:**

International guidelines use cancer antigen (CA) 125 thresholds to recommend which patients with pelvic masses should undergo evaluation by gynecologic oncologists for ovarian cancer. However, CA-125 thresholds were developed from White populations. If CA-125 levels differ among patient populations, current guidelines may contribute to delayed ovarian cancer diagnoses among women of other races and ethnicities than White.

**Objective:**

To examine CA-125 levels at ovarian cancer diagnosis by patient race and ethnicity and associations of elevated CA-125 levels with timely treatment.

**Design, Setting, and Participants:**

This retrospective cohort study included all patients with ovarian cancer diagnosed between January 1, 2004, and December 31, 2020, using the US National Cancer Database. The data analysis was performed between November 1, 2023, and July 10, 2024.

**Exposure:**

Patient race and ethnicity as identified in the National Cancer Database.

**Main Outcome and Measures:**

Cancer antigen 125 level was defined as elevated or borderline and negative or normal. Multivariable logistic regression models were used to examine the association of patient race and ethnicity with CA-125 level overall and for epithelial and high-grade serous cancers. Generalized linear models were used to examine the association of CA-125 level with days from diagnosis to chemotherapy start for patients with stage II to IV ovarian cancer.

**Results:**

Of the 250 749 patients with ovarian cancer diagnosed between 2004 and 2020 (median [IQR] age, 62.0 [52.0-73.0] years; 0.4% American Indian, 3.7% Asian, 8.6% Black, 85.2% White, and 2.0% other or unknown race and 6.7% Hispanic, 88.8% non-Hispanic, and 4.6% of unknown ethnicity), 212 477 had measured CA-125 levels, and 88.2% had an elevated CA-125 level at diagnosis. Patients with American Indian, Asian, or Black race were less likely to have an elevated CA-125 level at ovarian cancer diagnosis than White patients. In multivariable analyses adjusted for stage, comorbidities, and menopausal status, Black patients had lower odds of elevated CA-125 levels (adjusted odds ratio [AOR], 0.77; 95% CI, 0.74-0.81) compared with White patients, as did American Indian patients (AOR, 0.77; 95% CI, 0.62-0.94). Among patients with high-grade serous ovarian cancer only, Black patients had a lower odds of having an elevated CA-125 level at diagnosis (AOR, 0.81; 95% CI, 0.73-0.91). Patients with stage II to IV ovarian cancer with false-negative CA-125 findings at diagnosis had 9.38 days longer (95% CI, 8.43-10.34 days) to chemotherapy start compared with patients with an elevated CA-125 level.

**Conclusions and Relevance:**

In this cohort study of patients with ovarian cancer, American Indian and Black patients were 23% less likely to have an elevated CA-125 level at diagnosis. Current CA-125 thresholds may miss racially and ethnically diverse patients with ovarian cancer. Work is needed to develop inclusive CA-125 thresholds and diagnostic guidelines and not compound disparities in ovarian cancer diagnosis and treatment.

## Key Points

**Question:**

What is the association of cancer antigen (CA) 125 levels at ovarian cancer diagnosis with patient race and ethnicity and time to start of treatment?

**Findings:**

In this cohort study of 212 477 patients with measured CA-125 levels, Black patients had a significantly lower odds of having an elevated CA-125 level at ovarian cancer diagnosis compared with White patients after adjusting for stage, comorbidities, and menopausal status. Patients with false-negative CA-125 findings started chemotherapy 9 days later than patients with elevated CA-125 levels.

**Meaning:**

These findings suggest that current CA-125 thresholds may miss Black patients with ovarian cancer and may delay timely treatment.

## Teaser

This cohort study uses National Cancer Database data to examine cancer antigen (CA) 125 levels at ovarian cancer diagnosis by patient race and ethnicity and whether elevated CA-125 levels are associated with timely treatment initiation.

## Introduction

First described in 1981, cancer antigen (CA) 125 in ovarian cancer was the first biomarker for cancer diagnosis and surveillance approved by the US Food and Drug Administration.^1,2^ Cancer antigen 125 is produced by ovarian cancer cells and detectable in the serum through a blood draw. Since ovarian cancer symptoms (eg, bloating, early satiety) can be subtle and attributed to benign conditions, the discovery of CA-125 was heralded as a major breakthrough to diagnose and triage suspected ovarian cancer. An early-stage ovarian cancer diagnosis has been associated with considerably longer survival. Triage to and treatment by a gynecologic oncologist have been associated with improved survival in ovarian cancer, even with late-stage disease.^3,4,5^

Cancer antigen 125 has accordingly been incorporated into national and international guidelines for the diagnosis of ovarian cancer, including a set of thresholds for triage of adnexal masses. The UK’s National Institute for Health and Care Excellence and the American College of Obstetrics and Gynecology have included CA-125 level in their suspected cancer pathways since 2011^6^ and 2007, respectively.^7^ The current American College of Obstetrics and Gynecology guideline for adnexal masses states, “The combination of an elevated CA 125 level and a pelvic mass in a postmenopausal woman is highly suspicious for malignancy, and patients with these findings should be referred to or treated in consultation with a gynecologic oncologist.”^8^^(p5)^ In practice, these guidelines may be interpreted as to not refer patients with a nonelevated CA-125 level and pelvic mass to gynecologic oncology, as noted in multiple cohort studies on delays in ovarian cancer diagnosis.^9,10,11^ Almost all international guidelines, including those from South American and Asian countries, use the CA-125 threshold of 35 U/mL or greater (to convert to kU/L, multiply by 1.0) as elevated and for referral to gynecologic oncology in accordance with the original 1981 study.^12^

The original studies of CA-125 were performed in Boston, Massachusetts, and validated in Minnesota.^2,13^ While race was not reported, the patient demographic in these locations during the study periods was predominantly White (80% in Boston and 98% in Minnesota).^14^ Race is a social construct and not a proxy for genetic variation or ancestry. Given that the majority of patients with ovarian cancer worldwide are of races and ethnicities other than White, validation of biomarkers in ancestrally diverse populations is essential to ensure that they do not perpetuate disparities created by structural racism. Cancer antigen 125 levels have been shown to be 10% to 37% lower in healthy Black women^15,16,17^ and up to 20% lower in Native American women^16,18^ than non-Hispanic White women. If CA-125 levels also differ among patients with cancer, current guidelines on referral to gynecologic oncology for elevated CA-125 levels may contribute to missed or delayed ovarian cancer diagnoses among women with racial and ethnic backgrounds other than White. Our objective for this study was to examine CA-125 levels at ovarian cancer diagnosis by patient race and ethnicity and the associations of elevated CA-125 level with time to treatment.

## Methods

This retrospective cohort study was determined by the University of Pennsylvania Institutional Review Board to be exempt from review and informed consent as data were deidentified and publicly available through the National Cancer Database (NCDB). We followed the Strengthening the Reporting of Observational Studies in Epidemiology (STROBE) reporting guideline.

All patients with ovarian cancer diagnosed between January 1, 2004, and December 31, 2020, in the US were included. We used hospital-reported data from NCDB, a joint program of the American College of Surgeons Commission on Cancer and the American Cancer Society. The Commission on Cancer’s NCDB and its participating hospitals are the sources of the deidentified data used in this study; they have not verified and are not responsible for the statistical validity of the data analysis or our conclusions. The NCDB collects standardized data from more than 1500 accredited hospitals and includes more than 70% of new cancer diagnoses in the US.^19^

Our primary outcome was elevated CA-125 level at ovarian cancer diagnosis. Cancer antigen 125 level was defined as elevated or borderline and negative or normal by each site prior to treatment start.^20^ The NCDB suggests that sites use the standard definition of CA-125 level being elevated at 35 U/mL or greater and negative or normal at lower values.^8,20^ We included a small number of patients (n = 400) with borderline CA-125 level as positive and elevated. Cancer antigen 125 reporting was optional in 2004-2017 and became mandatory in 2018-2020, so we performed sensitivity analyses by these periods and with the removal of borderline CA-125 values.

Our secondary outcome was time to chemotherapy initiation after pathologic diagnosis for patients with stage II to IV ovarian cancer. National and international guidelines recommend chemotherapy for patients with stage II to IV ovarian cancer across histology types.^21^

We constructed a multivariable logistic regression model for the primary outcome of elevated CA-125 level at diagnosis. The model included clinically significant variables available to clinicians at the time of ovarian cancer diagnosis (menopausal status, comorbidities, and stage) and race and ethnicity. We reported odds ratios (ORs) for all histologies of ovarian cancers, for epithelial tumors, and for high-grade serous tumors. Histology codes were identified using the *International Classification of Diseases for Oncology, Third Edition* (eTable 1 in Supplement 1).^22,23^

For the secondary outcome of time to chemotherapy, we used a general linearized model for clinically relevant variables and a second model that included these variables and all available variables in the NCDB associated with cancer care delivery. The second model included the aforementioned clinically relevant covariates and additional variables known to influence access to care (insurance, census region, distance traveled for care, care at an academic center, median household income, and education). Time was calculated from the date of pathologic diagnosis to the date of first chemotherapy administration in days. Normal error distribution and identity link were used in both models. We performed an overall analysis of time to chemotherapy and then stratified by elevated CA-125 level.

We used standard NCDB definitions for covariates. For clinically relevant variables, race (American Indian, Asian, Black, White, and other race not Asian, Black, or White) and Hispanic ethnicity could be self-reported by patients or facility designated. We approximated postmenopausal status as age 55 years or older at cancer diagnosis. Comorbidities were defined using the NCDB’s simplified Charlson Comorbidity Index score (0, 1, ≥2), adjusted for the fact that all patients had cancer. We included stage, which is typically obtained at the time of surgery (ie, after ovarian cancer diagnosis), as a clinically relevant variable because CA-125 level is known to increase with ovarian cancer stage. The NCDB defines stage using the American Joint Committee on Cancer pathologic staging supplemented with clinical staging when surgery had not been performed.

Insurance was defined as the primary insurance a patient had at the time of diagnosis or treatment start. Household income was defined as the median household income in a patient’s zip code at the time of diagnosis using the American Community Survey and dividing into quartiles. Educational attainment was defined as the proportion of individuals who did not graduate high school in a patient’s zip code at the time of diagnosis using the American Community Survey. We used the most recent American Community Survey quartiles, supplemented by prior years if missing in analyses. Zip code–level income and education are highly correlated with patient-level values.^19,24^ Distance traveled for care was the straight line distance between 2 points from a patient’s zip code to their treating hospital; this measure underestimates distance traveled, as routes are rarely linear.

### Statistical Analysis

With the use of descriptive statistics, we report CA-125 levels by patient characteristics. We used multivariable logistic regression models to examine the association of patient characteristics with CA-125 level overall and for epithelial and high-grade serous cancers. We used generalized linear models to examine the association of CA-125 findings with time to chemotherapy start for patients with stage II to IV disease. We performed several sensitivity analyses of the association of race and ethnicity with CA-125 level at diagnosis, including (1) all available cancer care delivery variables, (2) age as a continuous variable, (3) no borderline elevated values, and (4) only years 2018-2020 during which CA-125 reporting was mandatory. A *P* ≤ .01 was considered to be statistically significant by χ^2^ test for categorical variables and analysis of variance for continuous variables. The data analysis was performed between November 1, 2023, and July 10, 2024, using SAS, version 9.4 (SAS Institute Inc).

## Results

Of the 250 749 patients with ovarian cancer diagnosed between 2004 and 2020 (median [IQR] age, 62.0 [52.0-73.0] years; 0.4% of American Indian, 3.7% Asian, 8.6% Black, 85.2% White, and 2.0% other or unknown race and 6.7% of Hispanic, 88.8% non-Hispanic, and 4.6% unknown ethnicity) (Table 1), 212 477 (84.7%) had CA-125 levels measured at diagnosis. Patients identified as being of a race or ethnicity other than White were less likely to have CA-125 levels measured at diagnosis. Measurement of CA-125 levels at diagnosis was also lower in younger patients, patients with Medicaid or Medicare insurance, patients without insurance, patients with lower education levels, and patients treated at academic centers (eTable 2 in Supplement 1).

**Table 1.** Characteristics of Patients With Ovarian Cancer Overall and by CA-125 Levels, 2004-2020

Characteristic	Patients, No. (%)				*P* value^a^
	Overall (n = 250 749)	CA-125 level			
		Normal (n = 25 072)	Elevated (n = 187 404)	Unknown (n = 38 272)	
Race					
American Indian	1009 (0.4)	127 (0.5)	689 (0.4)	193 (0.3)	<.001
Asian	10 449 (3.7)	1054 (4.2)	6884 (3.7)	2511 (3.8)	
Black	24 051 (8.6)	2284 (9.1)	15 123 (8.1)	6644 (10.0)	
White	237 476 (85.2)	21 129 (84.1)	161 201 (86.0)	55 146 (83.3)	
Other^b^	2860 (1.0)	271 (1.1)	1726 (0.9)	863 (1.3)	
Unknown	2845 (1.0)	251 (1.0)	1738 (0.9)	856 (1.3)	
Hispanic					
Hispanic	18 586 (6.7)	1773 (7.1)	11 568 (6.2)	5245 (7.9)	<.001
Non-Hispanic	247 401 (88.8)	22 264 (88.6)	167 530 (89.4)	57 607 (87.0)	
Unknown	12 703 (4.6)	1079 (4.3)	8263 (4.4)	3361 (5.1)	
Age, y					
Median (IQR)	62.0 (52.0-73.0)	58.0 (48.0-68.0)	63.0 (54.0-73.0)	61.0 (50.0-73.0)	<.001
Mean (SD)	61.7 (15.1)	57.2 (15.5)	62.9 (14.1)	60.2 (17.0)	
Postmenopausal status (aged ≥55 y)					
0	83 261 (29.9)	10 194 (40.6)	50 118 (26.7)	22 949 (34.7)	<.001
1	195 429 (70.1)	14 922 (59.4)	137 243 (73.3)	43 264 (65.3)	
Stage					
I	60 229 (21.6)	13 082 (52.1)	28 608 (15.3)	18 539 (28.0)	<.001
II	21 868 (7.8)	2748 (10.9)	14 019 (7.5)	5101 (7.7)	
III	100 003 (35.9)	4211 (16.8)	77 591 (41.4)	18 201 (27.5)	
IV	65 590 (23.5)	1722 (6.9)	50 216 (26.8)	13 652 (20.6)	
Unknown	30 998 (11.1)	3353 (13.3)	16 927 (9.0)	20 280 (9.5)	
Comorbidities, No.^c^					
0	219 879 (78.9)	20 246 (80.6)	145 812 (77.8)	53 821 (81.3)	<.001
1	42 706 (15.3)	3713 (14.8)	29 942 (16.0)	9051 (13.7)	
≥2	16 105 (5.8)	1157 (4.6)	11 607 (6.2)	3341 (5.0)	
Primary insurance					
Medicaid	19 340 (6.9)	1810 (7.2)	12 879 (6.9)	4651 (7.0)	<.001
Medicare	118 824 (42.6)	8292 (33.0)	84 051 (44.9)	26 481 (40.0)	
Private	124 379 (44.6)	13 747 (54.7)	80 545 (43.0)	30 087 (45.4)	
Uninsured	10 880 (3.9)	952 (3.8)	7395 (3.9)	2533 (3.8)	
Unknown	5267 (1.9)	315 (1.3)	2491 (1.3)	2461 (3.7)	
Median income, $					
<46 277	40 289 (14.5)	3369 (13.4)	26 621 (14.2)	10 299 (15.6)	<.001
46 227-57 856	54 043 (19.4)	4532 (18.0)	37 032 (19.8)	12 479 (18.8)	
57 857-74 062	60 284 (21.6)	5333 (21.2)	40 939 (21.9)	14 012 (21.2)	
≥74 063	95 956 (34.4)	9065 (36.1)	63 427 (33.9)	23 464 (35.4)	
Unknown	28 118 (10.1)	2817 (11.2)	19 342 (10.3)	5959 (9.0)	
Proportion with no high school degree, %					
<5.0	56 976 (20.4)	5553 (22.1)	38 624 (20.6)	12 799 (19.3)	<.001
5.0-9.0	73 403 (26.3)	6633 (26.4)	49 987 (26.7)	16 783 (25.3)	
9.1-15.2	68 988 (24.8)	5811 (23.1)	46 206 (24.7)	16 971 (25.6)	
≥15.3	51 281 (18.4)	4307 (17.1)	33 254 (17.7)	13 720 (20.7)	
Unknown	28 042 (10.1)	2812 (11.2)	19 290 (10.3)	5940 (9.0)	
Distance from site of care, miles					
No. of patients	250 749	22 314	168 144	60 291	<.001
Range	0.0-4967.2	0.0-2641.2	0.0-4961.2	0.0-4967.2	
Median (IQR)	11.7 (5.1-29.9)	11.6 (5.2-28.3)	12.0 (5.2-31.1)	10.8 (4.8-27.1)	
Mean (SD)	34.1 (112.5)	31.4 (102.0)	34.8 (115.0)	33.1 (108.8)	
Census region					
Midwest	62 386 (22.4)	5915 (23.6)	45 595 (24.3)	10 876 (16.4)	<.001
North	55 460 (19.9)	4943 (19.7)	37 554 (20.0)	12 963 (19.6)	
South	94 056 (33.7)	6911 (27.5)	61 985 (33.1)	25 160 (38.0)	
West	46 332 (16.6)	4104 (16.3)	32 688 (17.4)	9540 (14.4)	
Unknown	20 456 (7.3)	3243 (12.9)	9539 (5.1)	7674 (11.6)	
Facility type					
Academic	151 687 (54.4)	13 518 (53.8)	106 090 (56.6)	32 079 (48.4)	<.001
Community	106 547 (38.2)	8355 (33.3)	71 732 (38.3)	26 460 (40.0)	
Unknown	20 456 (7.3)	3243 (12.9)	9539 (5.1)	7674 (11.6)	

Abbreviation: CA, cancer antigen.

^a^ Significant differences were based on χ^2^ test for categorical variables and analysis of variance for continuous variables, comparing elevated with normal CA-125 levels.

^b^ Included all patients not identified as Asian, Black, or White in the National Cancer Database.

^c^ Based on Charlson-Deyo score.

Of the 212 477 patients with measured CA-125 levels, 88.2% had an elevated CA-125 level at diagnosis. The proportion of patients with elevated CA-125 levels increased as expected with stage at diagnosis (from 68.6% at stage I to 96.7% at stage IV) (eTable 4 in Supplement 1). The proportion of patients with elevated CA-125 levels at diagnosis was higher for those with high-grade serous ovarian cancer compared with all ovarian cancer histologies (93.2% vs 88.2%, respectively) and for postmenopausal compared with premenopausal status (90.2% vs 83.1%, respectively) (eTable 4 in Supplement 1). Black patients were more likely to be diagnosed at an advanced stage (III and IV) of ovarian cancer than White patients (66.0% vs 63.1%) (eTable 3 in Supplement 1). Yet, Black patients had lower rates of elevated CA-125 levels at each stage compared with White patients, with 10.9% of Black patients with stage III to IV ovarian cancer having a nonelevated CA-125 level compared with 8.2% of White patients (Table 2).

**Table 2.** Elevated CA-125 Level and Stage at Diagnosis by Race

Race and stage	CA-125 level	
	Elevated at ovarian cancer diagnosis, % (No.)^a^^,^^b^	Normal at diagnosis, % (No.)^b^
American Indian (n = 816)		
I	63.4 (123)	36.6 (71)
II	77.0 (47)	23.0 (14)
III	93.8 (304)	6.2 (20)
IV	98.3 (170)	1.7 (3)
Unknown	70.3 (45)	29.7 (19)
Asian (n = 7938)		
I	69.8 (1487)	30.2 (643)
II	84.3 (642)	15.7 (120)
III	95.1 (2567)	4.9 (133)
IV	97.6 (1626)	2.4 (40)
Unknown	82.6 (562)	17.4 (118)
Black (n = 17 407)		
I	63.1 (1829)	36.9 (1070)
II	82.1 (934)	17.9 (204)
III	93.6 (5544)	6.4 (376)
IV	95.5 (5318)	4.5 (248)
Unknown	79.5 (1498)	20.5 (386)
White (n = 182 330)		
I	69.0 (24 541)	31.0 (11 025)
II	83.7 (12 098)	16.3 (2360)
III	95.0 (67 866)	5.0 (3597)
IV	96.8 (42 213)	3.2 (1401)
Unknown	84.1 (14 483)	15.9 (2746)
Other (n = 1997)^c^		
I	69.5 (328)	30.5 (144)
II	87.6 (162)	12.4 (23)
III	92.9 (658)	7.1 (50)
IV	96.3 (412)	3.7 (16)
Unknown	81.4 (166)	18.6 (38)
Unknown (n = 1989)		
I	69.9 (300)	30.1 (129)
II	83.4 (136)	16.6 (27)
III	94.9 (652)	5.1 (35)
IV	97.1 (477)	2.9 (14)
Unknown	79.0 (173)	21.0 (46)

Abbreviation: CA, cancer antigen.

^a^ Elevated CA-125 level includes patients reported with elevated and borderline values.

^b^ Percentages add to 100 across rows.

^c^ Included all patients not identified as Asian, Black, or White in the National Cancer Database.

Black patients were less likely to have an elevated CA-125 level at diagnosis when adjusted for race and ethnicity and clinically significant variables of menopausal status, stage, and comorbidities (Table 3). Compared with White patients, Black patients were less likely to have an elevated CA-125 level for all histologies of ovarian cancer (adjusted OR [AOR], 0.77; 95% CI, 0.74-0.81), for epithelial ovarian cancer (AOR, 0.86; 95% CI, 0.81-0.91), and for high-grade serous cancer (AOR, 0.81; 95% CI, 0.73-0.91). American Indian patients were less likely to have an elevated CA-125 level for all ovarian cancer histologies (AOR, 0.77; 95% CI, 0.62-0.94); this difference was not present for epithelial subgroups, which had small sample sizes. Asian patients were more likely to have an elevated CA-125 level at diagnosis than White patients among those with high-grade serous cancer (AOR, 1.32; 95% CI, 1.10-1.57).

**Table 3.** Odds of Elevated CA-125 Level at Ovarian Cancer Diagnosis, 2004-2020^a^

Variable	Ovarian cancer type					
	All (n = 212 477)		Epithelial (n = 201 146)		High-grade serous (n = 76 784)	
	AOR (95% CI)	*P* value	AOR (95% CI)	*P* value	AOR (95% CI)	*P* value
Race						
American Indian	0.77 (0.62-0.94)	.01	0.77 (0.62-0.95)	.02	0.78 (0.51-1.19)	.25
Asian	1.07 (1.00-1.20)	.06	1.05 (0.98-1.13)	.17	1.32 (1.10-1.57)	.002
Black	0.77 (0.74-0.81)	<.001	0.86 (0.81-0.91)	<.001	0.81 (0.73-0.91)	<.001
White	1 [Reference]	NA	1 [Reference]	NA	1 [Reference]	NA
Other^b^	1.00 (0.87-1.15)	.99	0.98 (0.85-1.14)	.81	0.92 (0.70-1.22)	.58
Unknown	0.98 (0.85-1.14)	.80	1.00 (0.86-1.17)	.96	0.99 (0.73-1.34)	.94
Hispanic ethnicity						
Hispanic	0.94 (0.89-1.00)	.04	0.99 (0.93-1.05)	.76	1.02 (0.90-1.16)	.73
Non-Hispanic	1 [Reference]	NA	1 [Reference]	NA	1 [Reference]	NA
Unknown	1.01 (0.94-1.09)	.76	1.00 (0.93-1.08)	.96	0.99 (0.85-1.16)	.92
Menopausal status						
Aged <55 y	0.82 (0.79-0.84)	<.001	0.89 (0.86-0.92)	<.001	0.99 (0.92-1.06)	.72
Aged ≥55 y	1 [Reference]	NA	1 [Reference]	NA	1 [Reference]	NA
Stage						
I	0.08 (0.07-0.08)	<.001	0.08 (0.07-0.08)	<.001	0.08 (0.07-0.09)	<.001
II	0.17 (0.16-0.19)	<.001	0.18 (0.17-0.19)	<.001	0.16 (0.14-0.18)	<.001
III	0.64 (0.60-0.67)	<.001	0.63 (0.60-0.67)	<.001	0.61 (0.56-0.67)	<.001
IV	1 [Reference]	NA	1 [Reference]	NA	1 [Reference]	NA
Unknown	0.18 (0.17-0.19)	<.001	0.18 (0.17-0.19)	<.001	0.29 (0.25-0.33)	<.001
Comorbidities^c^						
0	0.85 (0.80-0.91)	<.001	0.87 (0.81-0.93)	<.001	0.94 (0.83-1.08)	.39
1	0.87 (0.81-0.94)	<.001	0.89 (0.82-0.96)	.003	0.93 (0.80-1.07)	.32
≥2	1 [Reference]	NA	1 [Reference]	NA	1 [Reference]	NA

Abbreviations: AOR, adjusted odds ratio; CA, cancer antigen; NA, not applicable.

^a^ All results were adjusted for race and ethnicity, postmenopausal status, stage at diagnosis, and comorbidities.

^b^ Included all patients not identified as Asian, Black, or White in the National Cancer Database.

^c^ Based on Charlson-Deyo score.

In the sensitivity analysis adjusted for clinically significant and sociodemographic variables (Figure; eTable 5 in Supplement 1), Black patients with ovarian cancer remained less likely to have an elevated CA-125 level at diagnosis (AOR, 0.72; 95% CI, 0.68-0.76) compared with White patients. Black patients were less likely to have an elevated CA-125 level at diagnosis of epithelial tumors (AOR, 0.78; 95% CI, 0.73-0.83) and high-grade serous tumors (AOR, 0.74; 95% CI, 0.77-0.84). American Indian patients were less likely to have an elevated CA-125 level for all ovarian cancer histologies (AOR, 0.73; 95% CI, 0.57-0.92), and Asian patients were more likely to have an elevated CA-125 level for high-grade serous ovarian cancer (AOR, 1.30; 95% CI, 1.07-1.59). There remained a consistent association of Black race with nonelevated CA-125 levels at ovarian cancer diagnosis when age was added as a continuous variable (eTable 6 in Supplement 1) and when the analysis was limited to 2018-2020, the period during which CA-125 reporting was mandatory (eTable 7 in Supplement 1). Findings remained the same when the 400 patients with borderline CA-125 elevation were removed from the analysis (eTable 8 in Supplement 1).

**Figure.**
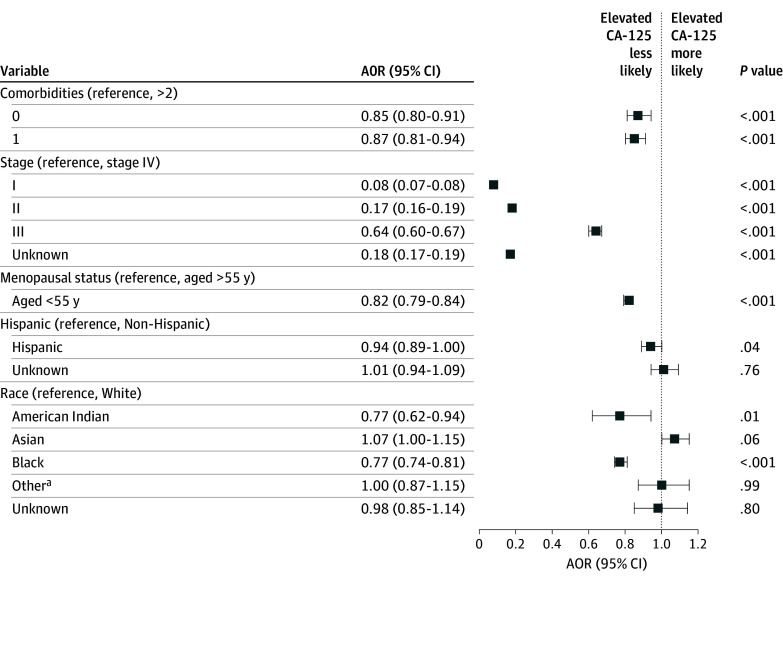
Association of Elevated Cancer Antigen (CA) 125 Level at Ovarian Cancer Diagnosis With Race and Ethnicity, 2004-2020 AOR indicates adjusted odds ratio. ^a^Other race included all patients who were not identified as Asian, Black, or White in the National Cancer Database.

Among the population of patients recommended to receive chemotherapy (stage II-IV), having an elevated CA-125 level was associated with 9.38 days shorter to chemotherapy initiation (95% CI, 8.43-10.34 days) (Table 4). Time to chemotherapy decreased as stage increased, with patients with stage II disease receiving chemotherapy 8.05 days later than those with stage IV disease (95% CI, 7.28-8.82 days). Black patients also experienced a longer time to chemotherapy initiation (3.26 days; 95% CI, 2.43-4.09 days), as did Hispanic patients (3.51 days; 95% CI, 2.52-4.51 days) compared with White patients. Patients with Medicaid experienced 2.58 more days (95% CI, 1.64-3.53 days) to treatment. When stratified by elevated CA-125 level, there remained slight differences in time to chemotherapy initiation by race: Black patients with an elevated CA-125 level had a 3.14-day delay in initiation (95% CI, 2.29-3.99 days) and with a nonelevated CA-125 level, a 4.99-day delay (95% CI, 1.15-8.84) in initiation compared with White patients.

**Table 4.** Time to Chemotherapy for Patients With Stage II to IV Ovarian Cancer, 2004-2020 (n = 100 863)

Variable	All patients		CA-125 level			
			Not elevated (n = 5522)		Elevated (n = 95 341)	
	Adjusted estimate (95% CI), d	*P* value	Estimate (95% CI), d	*P* value	Estimate (95% CI), d	*P* value
Elevated CA-125 level	−9.38 (−10.34 to −8.43)	<.001	NA	NA	NA	NA
Race						
American Indian	0.12 (−3.48 to 3.72)	.95	10.02 (−5.42 to 25.45)	.20	−0.54 (−4.24 to 3.16)	.77
Asian	−0.11 (−1.33 to 1.12)	.86	−2.77 (−8.75 to 3.22)	.36	0.07 (−1.18 to 1.32)	.91
Black	3.26 (2.43 to 4.09)	<.001	4.99 (1.15 to 8.84)	.01	3.14 (2.29 to 3.99)	<.001
White	1 [Reference]	NA	1 [Reference]	NA	1 [Reference]	NA
Other	−1.51 (−3.90 to 0.89)	.22	−5.10 (−16.11 to 5.90)	.36	−1.27 (−3.72 to 1.19)	.31
Unknown	1.23 (−1.22 to 3.68)	.33	3.82 (−8.29 to 15.92)	.54	1.07 (−1.43 to 3.57)	.40
Ethnicity						
Hispanic	3.51 (2.52 to 4.51)	<.001	3.54 (−1.17 to 8.25)	.14	3.53 (2.51 to 4.55)	<.001
Non-Hispanic	1 [Reference]	NA	1 [Reference]	NA	1 [Reference]	NA
Unknown	−1.09 (−2.17 to −0.01)	.05	−3.11 (−8.42 to 2.19)	.25	−0.99 (−2.09 to 0.10)	.08
Postmenopausal status						
Aged <55 y	0.93 (0.36 to 1.51)	.002	2.49 (−0.20 to 5.17)	.07	3.53 (2.51 to 4.55)	<.001
Aged ≥55 y	1 [Reference]	NA	1 [Reference]	NA	1 [Reference]	NA
Stage						
II	8.05 (7.28 to 8.82)	<.001	1.21 (−1.88 to 4.29)	.44	8.67 (7.86 to 9.48)	<.001
III	4.95 (4.48 to 5.42)	<.001	0.36 (−2.49 to 3.21)	.81	5.09 (4.62 to 5.57)	<.001
IV	1 [Reference]	NA	1 [Reference]	NA	1 [Reference]	NA
Comorbidities						
0	0.02 (−0.93 to 0.98)	.96	−2.24 (−7.16 to 2.69)	.37	0.15 (−0.82 to 1.12)	.76
1	−0.09 (−1.15 to 0.98)	.87	−3.48 (−8.87 to 1.91)	.21	0.11 (−0.98 to 1.19)	.84
≥2	1 [Reference]	NA	1 [Reference]	NA	1 [Reference]	NA
Insurance type						
Private	1 [Reference]	NA	1 [Reference]	NA	1 [Reference]	NA
Unknown	−0.89 (−2.87 to 1.10)	.38	2.71 (−7.43 to 12.85)	.60	−1.09 (−3.11 to 0.93)	.29
Medicaid	2.58 (1.64 to 3.53)	<.001	6.37 (1.70 to 11.04)	<.001	2.38 (1.42 to 3.34)	<.001
Medicare or other government	0.52 (0.03 to 1.02)	.04	2.50 (0.08 to 4.91)	.04	0.40 (−0.10 to 0.91)	.12
Uninsured	3.22 (1.99 to 4.45)	<.001	4.06 (−2.96 to 11.09)	.26	3.17 (1.92 to 4.41)	<.001
Median income, $						
<46 277	1.81 (0.98 to 2.64)	<.001	3.50 (−0.51 to 7.50)	.09	1.71 (0.86 to 2.56)	<.001
46 227-57 856	1.32 (0.63 to 2.00)	<.001	0.26 (−3.08 to 3.60)	.88	1.36 (0.67 to 2.06)	<.001
57 857-74 062	0.71 (0.10 to 1.32)	.02	−1.43 (−4.38 to 1.53)	.34	0.82 (0.20 to 1.44)	.01
≥74 063	1 [Reference]	NA	1 [Reference]	NA	1 [Reference]	NA
No high school degree, %						
<5.0	−0.66 (−1.51 to 0.19)	.17	−0.22 (−4.32 to 3.89)	.92	−0.67 (−1.53 to 0.20)	.13
5.0-9.0	0.17 (−0.57 to 0.92)	.65	2.44 (−1.23 to 6.11)	.19	0.05 (−0.71 to 0.81)	.89
9.1-15.2	0.38 (−0.31 to 1.07)	.28	3.07 (−0.30 to 6.44)	.08	0.24 (−0.46 to 0.94)	.51
≥15.3	1 [Reference]	NA	1 [Reference]	NA	1 [Reference]	NA
Census region						
West	1 [Reference]	NA	1 [Reference]	NA	1 [Reference]	NA
Midwest	−2.80 (−3.50 to −2.10)	<.001	−4.93 (−8.24 to −1.62)	<.001	−2.66 (−3.37 to −1.94)	<.001
North	−0.23 (−0.94 to 0.47)	.52	−3.16 (−6.49 to 0.17)	.06	−0.03 (−0.76 to 0.69)	.93
South	−2.72 (−3.38 to −2.06)	<.001	−6.06 (−9.18 to −2.95)	<.001	−2.52 (−3.19 to −1.85)	<.001
Facility type						
Community	1 [Reference]	NA	1 [Reference]	NA	1 [Reference]	NA
Academic	−0.11 (−0.55 to 0.34)	.64	−0.49 (−2.69 to 1.71)	.66	−0.10 (−0.55 to 0.36)	.68
Distance traveled for care	0	.04	0.00 (−0.01 to 0.01)	.45	0	.02

Abbreviations: CA, cancer antigen; NA, not applicable.

^a^ Reported estimates adjusted for all presented variables.

^b^ Included all patients not identified as Asian, Black, or White in the National Cancer Database.

## Discussion

In this NCDB-based cohort study of patients with ovarian cancer, Black patients were 23% less likely to have an elevated CA-125 level at ovarian cancer diagnosis than White patients. American Indian patients were also 23% less likely to have an elevated CA-125 level at diagnosis. Patients with a nonelevated CA-125 level started chemotherapy 9 days later on average. With CA-125 currently integrated into international guidelines for workup of adnexal masses, this difference in false-negative CA-125 rates by race may lead to underworkup and underreferral of American Indian and Black patients, contributing to a later-stage diagnosis of ovarian cancer in these patients. With 19 000 new ovarian cancer diagnoses annually in the US and 314 000 worldwide, if the CA-125 thresholds were updated to have similar sensitivity for Black patients as White patients, we estimate that at least 60 patients each year would be diagnosed at an earlier stage in the US and 1500 worldwide.^25,26^

Our findings of a lower CA-125 level at ovarian cancer diagnosis in Black patients is consistent with studies of the association of race and CA-125 levels in healthy women. Studies have consistently shown anywhere from a 10% to 29% difference in mean CA-125 values between these 2 populations in both premenopausal and postmenopausal women. Three cohort studies (2 from the US and 1 from the UK) found lower CA-125 levels in Black postmenopausal women,^15,16,17^ and 1 cohort study found lower CA-125 levels in Black premenopausal women^27^; all 4 studies had White women as the comparator group. Patterns of genetic variation are complex, and ancestry is not reported in the NCDB. We anticipated that the observed differences in CA-125 levels at ovarian cancer diagnosis would vary within and across populations by ancestry, which is why inclusive levels that are applicable and sensitive across genetically diverse populations are needed.

The rates of elevated CA-125 levels we found by stage at diagnosis are similar to prior studies. Given the known false-negative finding of CA-125 for patients with stage I ovarian cancer (hence its failure as a screening biomarker), 4 recent studies, 2 in ovarian cancer screening and 2 in patients exhibiting symptoms, have proposed lower thresholds for elevated CA-125 level. The UK Collaborative Trial of Ovarian Cancer Screening reported that sensitivity increased from 41.3% to 66.5% with a CA-125 threshold of 22 U/mL, albeit with a higher need for repeat screening.^28^ The UK Familial Ovarian Cancer Screening Study used thresholds of 35 U/mL in premenopausal and 30 U/mL in postmenopausal patients.^29^ For patients exhibiting symptoms undergoing workup for ovarian cancer, the Diagnosing Ovarian Cancer Early study in Canada used a CA-125 threshold of 25 U/mL.^30^ Patients in these UK and Canadian studies were predominantly White (UK Collaborative Trial of Ovarian Cancer Screening, 96.4%; Diagnosing Ovarian Cancer Early, 6.2%; UK Familial Ovarian Cancer Screening, not reported). A study of patients exhibiting symptoms in China proposed an even lower CA-125 threshold of 7.65 U/mL.^31^

One potential mechanism for differences in CA-125 thresholds is that CA-125 is an epitope of MUC-16, a much larger glycoprotein expressed on neutrophils.^32^ Thus, CA-125 level is correlated with neutrophil count in women with cancer^33^ across histology types^34,35^ and in women without cancer.^15^ At the cellular level, the expression levels of MUC-16 in fallopian tubes with benign tumors are similar between women who identify as Black and as White (Ronny Drapkin, MD, University of Pennsylvania Perelman School of Medicine, email, April 1, 2024). One potential mechanism for the differences seen in serum CA-125 levels may be benign ethnic neutropenia, also known as Duffy null phenotype. In benign ethnic neutropenia, the absolute neutrophil count is less than 1500/μL without an increased risk of infection. Neutrophils account for 40% to 80% of white blood cells, so a low white blood cell count often is used as a proxy.^36^ Benign ethnic neutropenia is most common in individuals of African, Caribbean, Middle Eastern, and West Indian descent and can be up to 50% in some populations. Some countries have adapted their normal absolute neutrophil count ranges to account for this benign genetic variation.^36^ Recognition of benign ethnic neutropenia has led to recalibration of absolute neutrophil count thresholds for clinical trials to promote inclusivity.^37^ Populations with a higher rate of benign ethnic neutropenia may have lower CA-125 levels with or without a cancer diagnosis, which may have contributed to the false-negative findings in our study. Further work is needed to develop inclusive CA-125 thresholds and guidelines for ovarian cancer diagnoses that are valid in diverse populations.

People who identify as multiracial are the largest growing segment of the US population, so developing a CA-125 threshold with similar sensitivity across populations is essential. Studies of ovarian cancer screening using CA-125 levels could be reanalyzed to see whether a lower CA-125 threshold might improve sensitivity and stage shift as seen in the UK Collaborative Trial of Ovarian Cancer Screening.^28^ A 9-day difference in time to chemotherapy initiation for patients with false-negative CA-125 findings may keep some patients within the 42-day recommended window between surgery and chemotherapy start but has the potential to represent a clinically meaningful delay.^38^

### Limitations

There are limitations to our study. The NCDB includes 80% of new ovarian cancer diagnoses in the US but does not include patients living in Puerto Rico, those treated by the Veterans Health Administration, or those cared for in small, non–National Cancer Institute–designated cancer centers. Patients in the NCDB may be less ethnically diverse than the full population of patients with ovarian cancer in the US and worldwide, which may bias the findings toward the null. The various NCDB sites’ definitions of an elevated CA-125 level may differ, although we did not anticipate that any sites used a lower threshold than 35 U/mL. The NCDB does not include ancestry. Race is a social construct and should not be construed as a proxy for ancestry or genetics in this analysis, and race-based thresholds should not be considered a step forward.

## Conclusions

In this cohort study of patients with ovarian cancer identified from the NCDB, American Indian and Black patients were 23% less likely to have an elevated CA-125 level at ovarian cancer diagnosis than White patients. Further work is needed to develop inclusive CA-125 thresholds and guidelines for an ovarian cancer diagnosis and prevent compounding disparities.

## Notes

Supplement 1. **eTable 1.** Histology Classifications of Ovarian Cancer **eTable 2.** CA-125 Measurement by Patient Sociodemographics **eTable 3.** Stage at Diagnosis by Histology and Race **eTable 4.** CA-125 Elevation at Diagnosis by Stage, Race, and Menopausal Status **eTable 5.** CA-125 Elevation Regression Analysis for All Variables, 2004-2020 **eTable 6.** Full Model for CA-125, Including Age as a Continuous Variable, 2004-2020 **eTable 7.** Full Model for CA-125 During the Period When CA-125 Reporting Was Required, 2018-2020 **eTable 8.** Full Model for CA-125, Excluding 400 Patients With Borderline CA-125 Elevation

Supplement 2.
Data Sharing Statement
