# Supplementary material for: Cancer Antigen 125 Levels at Time of Ovarian Cancer Diagnosis by Race and Ethnicity
Source: JAMA Netw Open. 2025 Mar 20;8(3):e251292. doi: 10.1001/jamanetworkopen.2025.1292 (PMC11926651; doi:10.1001/jamanetworkopen.2025.1292)
Supplement: Supplement 1. — eTable 1. Histology Classifications of Ovarian Cancer eTable 2. CA-125 Measurement by Patient Sociodemographics eTable 3. Stage at Diagnosis by Histology and Race eTable 4. CA-125 Elevation at Diagnosis by Stage, Race, and Menopausal Status eTable 5. CA-125 Elevation Regression Analysis for All Variables, 2004-2020 eTable 6. Full Model for CA-125, Including Age as a Continuous Variable, 2004-2020 eTable 7. Full Model for CA-125 During the Period When CA-125 Reporting Was Required, 2018-2020 eTable 8. Full Model for CA-125, Excluding 400 Patients With Borderline CA-125 Elevation [file jamanetwopen-e251292-s001.pdf]

Supplemental Online Content

Bodurtha Smith AJ, Gleason E, Kadiyala S, Wang X, Howell EA, McCarthy AM. Cancer antigen 125 levels at time of ovarian cancer diagnosis by race and ethnicity. *JAMA Netw Open*. 2025;8(3):251292. doi:10.1001/jamanetworkopen.2025.1292

eTable 1. Histology Classifications of Ovarian Cancer

eTable 2. CA-125 Measurement by Patient Sociodemographics

eTable 3. Stage at Diagnosis by Histology and Race

eTable 4. CA-125 Elevation at Diagnosis by Stage, Race, and Menopausal Status

eTable 5. CA-125 Elevation Regression Analysis for All Variables, 2004-2020

eTable 6. Full Model for CA-125, Including Age as a Continuous Variable, 2004-2020

eTable 7. Full Model for CA-125 During the Period When CA-125 Reporting Was Required, 2018-2020

eTable 8. Full Model for CA-125, Excluding 400 Patients With Borderline CA-125 Elevation

This supplemental material has been provided by the authors to give readers additional information about their work.

© 2025 Bodrutha Smith AJ et al. *JAMA Network Open*.

eTable 1. Histology Classifications of Ovarian Cancer		
**Histological group**	**Histological subtype**	**ICD-O-3 morphology code**
Epithelial tumors	Clear cell carcinoma	8005, 8310, 8313, 8443
	Endometrioid carcinoma	8380, 8381, 8382, 8383
	Mucinous carcinoma	8470, 8471, 8472, 8473, 8474, 8480, 8481, 8482, 8490, 9015
	Low-grade serous or borderline	8442, 8451, 8460, 8462
	High-grade serous	8441, 8461, 8450, 9014
	Squamous carcinoma	8050, 8051, 8052, 8070, 8071, 8072, 8073, 8074, 8075, 8076, 8078, 8084, 8081, 8082, 8083, 8084
	Transitional cell, Brenner carcinoma	8120-8131, 9000
	Carcinoid tumor	8240, 8241, 8242, 8243, 8244, 8245, 8246, 8249
	Carcinosarcoma	8950, 8951, 8980, 8981, 8982
	Undifferentiated or other epithelial	8002, 8003, 8004, 8010, 8011, 8012, 8013, 8014, 8015, 8020, 8021, 8022, 8030, 8031, 8032, 8033, 8034, 8034, 8041, 8044, 8046, 8090-8110, 8140, 8141, 8143, 8147, 8230, 8231, 8255, 8260, 8261, 8262, 8263, 8320, 8323, 8300, 8311-8312, 8314-8322, 8324-8325, 8336-8337, 8341-8375, 8384-8440, 8452-8454, 8510, 8560, 8562, 8570, 8571, 8572, 8573,8574, 8575, 8500-8551, 8561-8562, 8589, 9110, 9111, 8313, 8381, 8930-8991, 9010-9030
Sarcoma	Sarcoma	8800, 8801, 8802, 8803, 8804, 8805, 8806, 8810, 8811, 8813, 8814, 8815, 8825, 8840, 8890, 8891, 8894, 8895, 8896, 8930, 8931, 8933
Germ cell	Germ cell	8330-8335, 8340, 9060, 9064, 9065, 9070, 9071, 9072, 9080, 9081, 9082, 9083, 9084, 9085, 9090, 9100, 9101, 9105
Sex cord-stromal	Sex cord-stromal	8590, 8600, 8620, 8621, 8639, 8631, 8632, 8634, 8670, 8671, 8810
Other specific non-epithelial	Other specific non-epithelial	8680-8806, 9040-9055, 9120-9373, 9530-9589
Non-specific	Non-specific	8000, 8001
Histologic subtypes were based on the World Health Organization's International Classification of Diseases for Oncology, Third Edition.<br>References: Matsuo 2023; Matz 2017; https://seer.cancer.gov/icd-o-3/sitetype.icdo3.20220429.pdf		

© 2025 Bodrutha Smith AJ et al. *JAMA Network Open*.

eTable 2. CA-125 Measurement by Patient Sociodemographics				
		**Overall (N=232,206)**		
		**% CA-125 Measured (N)**	**Adjusted OR (95%CI)**	**p-value**
Race	White	76.8 (182,330)	Reference	
	American Indian	80.1 (816)	0.66 (0.54, 0.79)	<.001
	Asian	76.0 (7938)	0.63 (0.59, 0.66)	<.001
	Black	72.4 (17,407)	0.85 (0.82, 0.89)	<.001
	Another race	69.8 (1997)	0.53 (0.48, 0.59)	<.001
	Unknown race	69.9 (1989)	0.64 (0.56, 0.73)	<.001
Hispanic ethnicity	Non-Hispanic	76.7 (189,794)	Reference	<.001
	Hispanic	71.8 (13,341)	0.77 (0.73, 0.81)	<.001
	Unknown	73.5 (9342)	3.50 (3.19, 3.84)	<.001
Postmenopausal	Age <55 years	72.4 (60,312)	Reference	<.001
	Age ≥ 55 years	77.9 (152,165)	1.20 (1.16, 1.24)	<.001
Stage	Unknown stage	65.4 (20,280)	1.02 (0.98, 1.06)	0.32
	Stage I	69.2 (41,690)	585 (0.00, 3E37)	0.88
	Stage II	76.7 (16,767)	1.20 (1.16, 1.25)	<.001
	Stage III	81.8 (81,802)	1.44 (1.37, 1.52)	<.001
	Stage IV	79.2 (51,938)	1.37 (1.33, 1.42)	<.001
Co-morbidities	0	75.5 (166,058)	Reference	
	1	78.8 (33,655)	1.49 (1.42, 1.55)	<.001
	2+	79.2 (12,764)	1.61 (1.53, 1.70)	<.001
Insurance	Private insurance	75.8 (94,292)	Reference	
	Medicaid	75.9 (14,689)	0.75 (0.71, 0.78)	<.001
	Medicare or other government	77.7 (92,343)	0.94 (0.92, 0.97)	<.001
	Uninsured	76.7 (8347)	1.16 (1.08, 1.24)	<.001
	Unknown	53.2 (2806)	2.04 (1.81, 2.29)	<.001
Median Income Quartiles	>= $74,063	75.5 (72,492)	Reference	
	$46,227-$57,856	76.9 (41,564)	1.01 (0.97, 1.05)	0.68
	$57,857-$74,062	76.7 (46,272)	1.03 (0.99, 1.06)	0.14
	< $46,277	74.4 (29,990)	1.12 (1.06, 1.17)	<.001
	Unknown	78.8 (22,159)		
Percent No High School Degree Quartiles	>= 15.3%	73.2 (37,561)	Reference	
	5.0%-9.0%	77.1 (56,620)	0.89 (0.86, 0.93)	<.001
	9.1%-15.2%	75.4 (52,017)	0.96 (0.92, 0.99)	0.02
	< 5.0%	86.1 (44,177)	0.92 (0.88, 0.96)	<.001
	Unknown	78.8 (22,102)		
Census Region	West	79.4 (36,792)	Reference	
	Midwest	82.6 (51,510)	0.93 (0.90, 0.97)	<.001
	North	76.6 (42,497)	1.07 (1.03, 1.11)	<.001
	South	73.2 (68,896)	1.06 (1.02, 1.10)	<.001
	Unknown	62.5 (12,782)	**	**
Facility type	Community	78.9 (119,608)	Reference	
	Academic	75.1 (80,087)	0.84 (0.82, 0.86)	<.001
	Unknown	62.5 (12,782)	**	**
Reported odds ratios are adjusted for all presented variables.<br>Region and facility data is suppressed for younger patients in the dataset. Another race is a category from the National Cancer Database that includes all patients who do not identify as Asian, Black, or White.				

© 2025 Bodrutha Smith AJ et al. *JAMA Network Open*.

eTable 3. Stage at Diagnosis by Histology and Race							
		**White**	**Black**	**American Indian**	**Asian**	**Another race**	**Unknown race**
		**% (N)**	**% (N)**	**% (N)**	**% (N)**	**% (N)**	**% (N)**
All ovarian cancers (212,477)	Stage I	19.5 (35,566)	16.7 (2,899)	23.8 (194)	26.8 (2,130)	23.6 (472)	21.6 (429)
	Stage II	7.9 (14,458)	6.5 (1,138)	7.5 (61)	9.6 (762)	9.3 (185)	8.2 (163)
	Stage III	39.2 (71,463)	34.0 (5,920)	39.7 (324)	34.0 (2,700)	35.5 (708)	34.5 (687)
	Stage IV	23.9 (43,614)	32.0 (5,566)	21.2 (173)	21.0 (1,666)	21.4 (428)	24.7 (491)
	AJCC stage group unknown	9.4 (17,229)	10.8 (1,884)	7.8 (64)	8.6 (680)	10.2 (204)	11.0 (219)
Epithelial cancers (201,146)	Stage I	18.9 (32,765)	13.7 (2,120)	22.6 (174)	25.9 (1,935)	22.0 (403)	20.1 (373)
	Stage II	8.0 (13,924)	6.5 (1,006)	6.9 (53)	9.7 (726)	9.5 (174)	8.4 (157)
	Stage III	40.4 (70,105)	36.4 (5,640)	41.2 (317)	35.1 (2,617)	37.5 (685)	36.1 (670)
	Stage IV	24.2 (42,112)	34.0 (5,277)	21.8 (168)	21.4 (1,599)	22.3 (407)	25.6 (475)
	AJCC stage group unknown	8.5 (14,813)	9.5 (1,467)	7.4 (57)	7.8 (584)	8.7 (160)	9.8 (183)
High-grade serous cancers (76,784)	Stage I	7.6 (5,080)	7.2 (415)	9.0 (28)	8.1 (211)	7.0 (55)	8.5 (61)
	Stage II	7.1 (4,746)	6.1 (351)	5.2 (16)	7.7 (202)	10.6 (84)	7.1 (51)
	Stage III	52.6 (35,078)	47.2 (2,701)	57.1 (177)	50.7 (1,325)	50.1 (395)	51.3 (367)
	Stage IV	26.4 (17,591)	33.1 (1,896)	25.2 (78)	27.8 (727)	24.7 (195)	26.0 (186)
	AJCC stage group unknown	6.2 (4,137)	6.4 (364)	3.5 (11)	5.6 (146)	7.6 (60)	7.0 (50)
Another race is a category from the National Cancer Database that includes all patients who do not identify as Asian, Black, or White.							

© 2025 Bodrutha Smith AJ et al. *JAMA Network Open*.

eTable 4. CA-125 Elevation at Diagnosis by Stage, Race, and Menopausal Status		
	Elevated CA-125 at ovarian cancer diagnosis	Normal CA-125 at diagnosis
	% (N)	% (N)
**Stage**		
All ovarian cancers (n=212,477)	88.2 (187,361)	11.8 (25,116)
Stage I	68.6 (28,608)	31.4 (13,082)
Stage II	83.6 (14,019)	16.4 (2748)
Stage III	94.9 (77,591)	5.1 (4211)
Stage IV	96.7 (50,216)	3.3 (1722)
Stage unknown	83.5 (16,927)	16.5 (3353)
Epithelial cancers (n=201,146)	89.1 (179,255)	10.9 (21,891)
Stage I	70.3 (26,567)	29.7 (11,203)
Stage II	84.3 (13,519)	15.7 (2,521)
Stage III	95.1 (76,095)	4.9 (3939)
Stage IV	96.8 (48,461)	3.2 (1577)
Stage unknown	84.6 (14,613)	15.4 (2651)
High-grade serous cancers (n=76,784)	93.2 (71,536)	6.8 (5,248)
Stage I	73.4 (4292)	26.6 (1558)
Stage II	84.6 (4608)	15.4 (842)
Stage III	95.5 (38,222)	4.5 (1821)
Stage IV	97.2 (20,084)	2.8 (589)
Stage unknown	90.8 (4330)	9.2 (438)
**Race**		
All ovarian cancers		
American Indian (n=916)	84.4 (689)	15.6 (127)
Asian (n=7938)	86.7 (6884)	13.3 (1054)
Black (n=17,407)	86.9 (15,123)	13.1 (2284)
Another race (n=1997)	86.4 (1726)	13.6 (271)
Unknown race (n=1487)	87.4 (1738)	12.6 (251)
White (n=182,330)	88.4 (161,201)	11.6 (21,129)
Epithelial cancers (n=201,146)		
American Indian	85.7 (659)	14.3 (110)
Asian	87.6 (6,533)	12.4 (928)
Black	89.5 (13,878)	10.5 (1,632)
Another race	87.8 (1,605)	12.2 (224)
Unknown race	88.7 (1,648)	11.3 (210)
White	89.2 (154,932)	10.8 (18,787)
High-grade serous cancers (n=76,784)		
American Indian	91.6 (284)	8.4 (26)
Asian	94.6 (2,469)	5.4 (142)
Black	5,283 (92.2)	7.8 (444)
Another race	92.4 (729)	7.6 (60)
Unknown race	92.9 (664)	7.1 (51)
White	93.2 (62,107)	6.8 (4,525)
**Menopausal status**		
All ovarian cancers (n=212,477)		
Pre-menopausal: Age < 55 years	83.1 (50,118)	16.9 (10,194)
Postmenopausal: Age ≥ 55 years	90.2 (137,243)	9.8 (14,922)
Epithelial cancers (n=201,146)		
Pre-menopausal: Age < 55 years	85.1 (46,442)	14.9 (8,122)
Postmenopausal: Age ≥ 55 years	90.6 (132,813)	9.4 (13,769)
High-grade serous cancers (n=76,784)		
Pre-menopausal: Age < 55 years	92.3 (15,960)	7.7 (1,339)
Postmenopausal: Age ≥ 55 years	93.4 (55,576)	6.6 (3,909)
Elevated CA-125 includes patients reported with elevated and borderline values.<br>Another race is a category from the National Cancer Database that includes all patients who do not identify as Asian, Black, or White.		

© 2025 Bodrutha Smith AJ et al. *JAMA Network Open*.

eTable 5. CA-125 Elevation Regression Analysis for All Variables, 2004-2020							
		**Overall (N=178,498)**		**Epithelial Tumors (N=171,656)**		**High-grade Serous Tumors (N=65,945)**	
		**Adjusted OR (95%CI)**	**P-value**	**OR (95%CI)**	**P-value**	**Adjusted OR (95%CI)**	**P-value**
Race	White	Reference					
	American Indian	0.73 (0.57, 0.92)	<.001	0.74 (0.57, 0.94)	0.014	0.77 (0.47, 1.23)	0.27
	Asian	1.10 (1.01, 1.20)	0.02	1.10 (1.01, 1.20)	0.03	1.30 (1.07, 1.59)	<.001
	Black	0.72 (0.68, 0.76)	<.001	0.78 (0.73, 0.83)	<.0001	0.74 (0.66, 0.84)	<.001
	Another race	1.01 (0.86, 1.19)	0.90	1.01 (0.85, 1.21)	0.88	0.86 (0.64, 1.17)	0.35
	Unknown race	1.06 (0.90, 1.26)	0.47	1.07 (0.90, 1.28)	0.44	0.94 (0.69, 1.30)	0.72
Hispanic ethnicity	Non-Hispanic	Reference					
	Hispanic	0.88 (0.82, 0.94)	<.001	0.91 (0.84, 0.97)	0.008	0.97 (0.83, 1.12)	0.65
	Unknown	1.00 (0.93, 1.08)	0.96	0.99 (0.91, 1.07)	0.73	0.99 (0.84, 1.16)	0.87
Postmenopausal	Age <55 years	0.96 (0.93, 1.00)	0.05	1.00 (0.96, 1.05)	0.86	1.04 (0.96, 1.14)	0.32
	Age ≥ 55 years	Reference					
Stage	Unknown stage	0.19 (0.17, 0.20)	<.001	0.19 (0.18, 0.21)	<.0001	0.29 (0.25, 0.33)	<.001
	Stage I	0.08 (0.07, 0.08)	<.001	0.08 (0.07, 0.08)	<.0001	0.08 (0.07, 0.09)	<.001
	Stage II	0.18 (0.16, 0.19)	<.001	0.17 (0.16, 0.19)	<.0001	0.16 (0.14, 0.18)	<.001
	Stage III	0.64 (0.60, 0.68)	<.001	0.63 (0.59, 0.67)	<.0001	0.61 (0.55, 0.67)	<.001
	Stage IV	Reference					
Co-morbidities	0	0.90 (0.84, 0.97)	<.001	0.91 (0.84, 0.98)	0.011	0.92 (0.80, 1.07)	0.29
	1	0.90 (0.83, 0.97)	<.001	0.90 (0.83, 0.98)	0.011	0.88 (0.75, 1.04)	0.13
	2+	Reference					
Insurance	Private insurance	Reference					
	Medicaid	1.21 (1.13, 1.30)	<.0001	1.22 (1.13, 1.32)	<.001	1.23 (1.05, 1.43)	0.008
	Medicare or other government (e.g., VA, Tricare)	1.13 (1.09, 1.18)	<.0001	1.12 (1.08, 1.16)	<.001	1.04 (0.97, 1.12)	0.29
	Uninsured	1.43 (1.30, 1.56)	<.0001	1.44 (1.30, 1.58)	<.001	1.53 (1.22, 1.90)	0.0002
	Unknown	1.21 (1.05, 1.40)	0.008	1.22 (1.05, 1.42)	<.001	1.02 (0.76, 1.35)	0.91
Median Income Quartiles	>= $74,063	Reference					
	$46,227-$57,856	1.09 (1.04, 1.15)	0.0008	1.10 (1.04, 1.16)	<.001	1.06 (0.96, 1.18)	0.24
	$57,857-$74,062	1.05 (1.00, 1.10)	0.032	1.05 (1.00, 1.10)	0.04	1.08 (0.99, 1.18)	0.10
	< $46,277	1.04 (0.98, 1.11)	0.16	1.05 (0.98, 1.12)	0.17	1.04 (0.92, 1.18)	0.51
Percent No High School Degree Quartiles	>= 15.3%	Reference					

© 2025 Bodrutha Smith AJ et al. *JAMA Network Open*.

	5.0%-9.0%	0.98 (0.93, 1.03)	0.41	0.99 (0.93, 1.04)	0.62	0.98 (0.88, 1.10)	0.75
	9.1%-15.2%	0.99 (0.94, 1.04)	0.72	1.00 (0.95, 1.05)	1.00	1.05 (0.95, 1.17)	0.36
	< 5.0%	0.95 (0.89, 1.01)	0.077	0.94 (0.88, 1.01)	0.07	0.94 (0.83, 1.06)	0.32
Census Region	West	Reference					
	Midwest	1.00 (0.95, 1.05)	0.85	1.02 (0.96, 1.07)	0.57	0.99 (0.90, 1.10)	0.87
	North	0.97 (0.92, 1.02)	0.26	0.98 (0.93, 1.03)	0.42	0.94 (0.85, 1.04)	0.22
	South	1.09 (1.04, 1.14)	<.001	1.09 (1.04, 1.15)	<.001	1.03 (0.94, 1.14)	0.50
Facility Type	Community	Reference					
	Academic	0.98 (0.95, 1.01)	0.25	0.98 (0.95, 1.02)	0.35	0.99 (0.93, 1.06)	0.78
Distance traveled for care		1.00 (1.00, 1.00)	<.001	1.00 (1.00, 1.00)	<.001	1.00 (1.00, 1.00)	<.001
Reported odds ratios are adjusted for all presented variables. Numbers are smaller than Table 3 as patients with unknown variables for additional sociodemographics were removed from the analysis. Another race is a category from the National Cancer Database that includes all patients who do not identify as Asian, Black, or White.							

© 2025 Bodrutha Smith AJ et al. *JAMA Network Open*.

eTable 6. Full Model for CA-125, Including Age as a Continuous Variable, 2004-2020							
		**Overall (N= 178866)**		**Epithelial Tumors (N= 172002)**		**High-grade Serous Tumors (N= 66031**	
		**Adjusted OR (95%CI)**	**P-value**	**OR (95%CI)**	**P-value**	**Adjusted OR (95%CI)**	**P-value**
Race	White						
	American Indian	0.75 (0.59, 0.95)	0.02	0.76 (0.59, 0.97)	0.03	0.76 (0.47, 1.23)	0.26
	Asian	1.12 (1.03, 1.22)	0.01	1.11 (1.02, 1.21)	0.02	1.30 (1.07, 1.58)	0.01
	Black	0.72 (0.68, 0.77)	<.001	0.79 (0.74, 0.84)	<.001	0.74 (0.66, 0.84)	<.001
	Another race	1.02 (0.86, 1.20)	0.83	1.02 (0.86, 1.22)	0.82	0.87 (0.64, 1.18)	0.36
	Unknown race	1.07 (0.90, 1.27)	0.42	1.08 (0.90, 1.29)	0.40	0.94 (0.68, 1.29)	0.70
Hispanic ethnicity	Non-Hispanic						
	Hispanic	0.88 (0.82, 0.95)	<.001	0.91 (0.85, 0.98)	0.01	0.97 (0.84, 1.12)	0.65
	Unknown	1.00 (0.92, 1.08)	0.98	0.98 (0.91, 1.07)	0.69	0.99 (0.84, 1.17)	0.92
Postmenopausal	Age <55 years						
	Age ≥ 55 years	1.16 (1.10, 1.22)	<.001	1.17 (1.11, 1.23)	<.001	1.03 (0.92, 1.15)	0.64
Stage	Unknown stage	0.19 (0.17, 0.20)	<.001	0.19 (0.18, 0.21)	<.001	0.29 (0.25, 0.33)	<.001
	Stage I	0.08 (0.08, 0.08)	<.001	0.08 (0.08, 0.09)	<.001	0.08 (0.07, 0.09)	<.001
	Stage II	0.18 (0.17, 0.19)	<.001	0.18 (0.16, 0.19)	<.001	0.16 (0.14, 0.18)	<.001
	Stage III	0.64 (0.61, 0.69)	<.001	0.64 (0.60, 0.68)	<.001	0.61 (0.55, 0.67)	<.001
	Stage IV						
Co-morbidities	0	0.92 (0.85, 0.99)	0.02	0.92 (0.85, 0.99)	0.04	0.93 (0.80, 1.07)	0.30
	1	0.91 (0.84, 0.98)	0.02	0.91 (0.83, 0.98)	0.02	0.88 (0.75, 1.04)	0.13
	2+	Reference					
Insurance	Private insurance	Reference					
	Medicaid	1.21 (1.13, 1.30)	<.001	1.22 (1.13, 1.32)	<.001	1.22 (1.05, 1.42)	<.001
	Medicare or other government	0.99 (0.95, 1.04)	0.67	1.00 (0.95, 1.05)	0.97	1.05 (0.96, 1.15)	0.24
	Uninsured	1.42 (1.30, 1.56)	<.001	1.43 (1.30, 1.58)	<.001	1.52 (1.22, 1.90)	<.001
	Unknown	1.16 (1.01, 1.34)	0.04	1.18 (1.01, 1.37)	0.03	1.03 (0.77, 1.37)	0.86
Median Income Quartiles	>= $74,063						
	$46,227-$57,856	1.09 (1.04, 1.15)	<.001	1.10 (1.04, 1.16)	<.001	1.06 (0.96, 1.18)	0.24
	$57,857-$74,062	1.05 (1.00, 1.10)	0.03	1.05 (1.00, 1.10)	0.04	1.08 (0.99, 1.18)	0.10
	< $46,277	1.05 (0.98, 1.11)	0.15	1.05 (0.98, 1.12)	0.16	1.04 (0.92, 1.18)	0.50
Percent No High School Degree Quartiles	>= 15.3%	0.97 (0.92, 1.03)	0.35	0.98 (0.93, 1.04)	0.56	0.98 (0.88, 1.10)	0.74
	5.0%-9.0%						
	9.1%-15.2%	0.99 (0.94, 1.04)	0.68	1.00 (0.95, 1.05)	0.95	1.05 (0.94, 1.16)	0.40

© 2025 Bodrutha Smith AJ et al. *JAMA Network Open*.

	< 5.0%	0.94 (0.89, 1.00)	0.06	0.94 (0.88, 1.00)	0.06	0.94 (0.83, 1.06)	0.32
Census Region	West	Reference					
	Midwest	1.00 (0.95, 1.05)	0.85	1.02 (0.96, 1.07)	0.58	0.99 (0.89, 1.10)	0.85
	North	0.97 (0.92, 1.02)	0.21	0.98 (0.93, 1.03)	0.36	0.94 (0.85, 1.04)	0.22
	South	1.09 (1.04, 1.14)	<.001	1.09 (1.04, 1.15)	<.001	1.03 (0.94, 1.14)	0.50
Facility Type	Community	Reference					
	Academic	0.99 (0.96, 1.02)	0.47	0.99 (0.96, 1.02)	0.53	0.99 (0.93, 1.06)	0.80
Distance traveled for care		1.00 (1.00, 1.00)	<.001	1.00 (1.00, 1.00)	<.001	1.00 (1.00, 1.00)	<.001
Age (years)	cottage	1.01 (1.01, 1.02)	<.001	1.01 (1.01, 1.01)	<.001	1.00 (0.99, 1.00)	0.62
Reported odds ratios are adjusted for all presented variables. Another race is a category from the National Cancer Database that includes all patients who do not identify as Asian, Black, or White.							

© 2025 Bodrutha Smith AJ et al. *JAMA Network Open*.

eTable 7. Full Model for CA-125 During the Period When CA-125 Reporting Was Required, 2018-2020							
		**Overall (N= 30624)**		**Epithelial Tumors (N= 29449)**		**High-grade Serous Tumors (N= 15020)**	
		**Adjusted OR (95%CI)**	**P-value**	**OR (95%CI)**	**P-value**	**Adjusted OR (95%CI)**	**P-value**
Race	White						
	American Indian	0.97 (0.57, 1.66)	0.92	0.98 (0.55, 1.72)	0.94	1.37 (0.42, 4.47)	0.61
	Asian	1.06 (0.90, 1.26)	0.47	1.07 (0.90, 1.27)	0.46	1.21 (0.85, 1.72)	0.29
	Black	0.71 (0.63, 0.81)	<.001	0.80 (0.69, 0.92)	<.001	0.71 (0.56, 0.89)	<.001
	Another race	1.20 (0.88, 1.64)	0.26	1.14 (0.83, 1.58)	0.41	0.99 (0.58, 1.70)	0.97
	Unknown race	0.84 (0.58, 1.23)	0.37	0.82 (0.55, 1.21)	0.31	0.81 (0.40, 1.65)	0.57
Hispanic ethnicity	Non-Hispanic						
	Hispanic	0.86 (0.74, 0.99)	0.04	0.93 (0.80, 1.08)	0.35	1.06 (0.80, 1.39)	0.69
	Unknown	1.21 (0.88, 1.66)	0.25	1.23 (0.88, 1.72)	0.23	1.49 (0.80, 2.80)	0.21
Postmenopausal	Age <55 years						
	Age ≥ 55 years	0.97 (0.89, 1.06)	0.51	1.00 (0.91, 1.11)	0.92	1.11 (0.92, 1.34)	0.28
Stage	Unknown stage	0.20 (0.17, 0.23)	<.001	0.21 (0.18, 0.24)	<.001	0.35 (0.28, 0.45)	<.001
	Stage I	0.09 (0.08, 0.11)	<.001	0.09 (0.08, 0.11)	<.001	0.10 (0.08, 0.12)	<.001
	Stage II	0.17 (0.15, 0.20)	<.001	0.17 (0.15, 0.20)	<.001	0.16 (0.13, 0.20)	<.001
	Stage III	0.63 (0.55, 0.73)	<.001	0.63 (0.54, 0.72)	<.001	0.66 (0.54, 0.80)	<.001
	Stage IV	Reference					
Co-morbidities	0	0.92 (0.80, 1.05)	0.22	0.91 (0.78, 1.05)	0.20	0.88 (0.68, 1.14)	0.34
	1	0.88 (0.75, 1.04)	0.14	0.85 (0.72, 1.01)	0.06	0.78 (0.58, 1.05)	0.10
	2+	Reference					
Insurance	Private insurance	Reference					
	Medicaid	1.19 (1.03, 1.37)	0.02	1.22 (1.05, 1.42)	0.01	1.31 (0.97, 1.76)	0.08
	Medicare or other government	1.15 (1.05, 1.25)	<.001	1.14 (1.05, 1.25)	<.001	1.11 (0.96, 1.29)	0.16
	Uninsured	1.38 (1.10, 1.73)	<.001	1.31 (1.03, 1.67)	0.03	1.21 (0.76, 1.91)	0.42
	Unknown	1.15 (0.81, 1.64)	0.44	1.17 (0.80, 1.71)	0.41	1.64 (0.70, 3.83)	0.25
Median Income Quartiles	>= $74,063	Reference					
	$46,227-$57,856	1.02 (0.91, 1.14)	0.75	1.02 (0.90, 1.14)	0.80	1.03 (0.84, 1.27)	0.79
	$57,857-$74,062	1.08 (0.98, 1.20)	0.13	1.06 (0.96, 1.18)	0.26	1.08 (0.89, 1.30)	0.44
	< $46,277	0.99 (0.86, 1.14)	0.88	0.99 (0.86, 1.15)	0.92	1.08 (0.84, 1.40)	0.55
Percent No High School Degree Quartiles	>= 15.3%	Reference					
	5.0%-9.0%	0.88 (0.78, 1.00)	0.04	0.88 (0.77, 1.00)	0.06	1.05 (0.84, 1.32)	0.66

© 2025 Bodrutha Smith AJ et al. *JAMA Network Open*.

	9.1%-15.2%	0.94 (0.83, 1.05)	0.28	0.94 (0.83, 1.07)	0.35	1.19 (0.96, 1.47)	0.11
	< 5.0%	0.88 (0.77, 1.02)	0.08	0.89 (0.76, 1.03)	0.11	1.05 (0.82, 1.36)	0.68
Census Region	West	Reference					
	Midwest	0.98 (0.87, 1.10)	0.72	1.04 (0.92, 1.17)	0.55	1.03 (0.84, 1.27)	0.79
	North	1.02 (0.91, 1.15)	0.72	1.06 (0.94, 1.19)	0.36	1.02 (0.83, 1.25)	0.87
	South	1.16 (1.04, 1.29)	<.001	1.21 (1.08, 1.36)	<.001	1.12 (0.92, 1.36)	0.26
Facility Type	Community	Reference					
	Academic	0.92 (0.85, 1.00)	0.04	0.91 (0.84, 0.99)	0.03	0.90 (0.78, 1.03)	0.12
Distance Traveled for care		1.00 (1.00, 1.00)	0.18	1.00 (1.00, 1.00)	0.27	1.00 (1.00, 1.00)	0.37
Reported odds ratios are adjusted for all presented variables. Another race is a category from the National Cancer Database that includes all patients who do not identify as Asian, Black, or White.							

© 2025 Bodrutha Smith AJ et al. *JAMA Network Open*.

eTable 8. Full Model for CA-125, Excluding 400 Patients With Borderline CA-125 Elevation							
		**Overall (N= 178498)**		**Epithelial Tumors (N= 171656)**		**High-grade Serous Tumors (N= 65945)**	
		**Adjusted OR (95%CI)**	**P-value**	**Adjusted OR (95%CI)**	**P-value**	**Adjusted OR (95%CI)**	**P-value**
Race	White						
	American Indian	0.73 (0.57, 0.92)	<.001	0.74 (0.57, 0.94)	0.01	0.77 (0.47, 1.23)	0.27
	Asian	1.10 (1.01, 1.20)	0.02	1.10 (1.01, 1.20)	0.03	1.30 (1.07, 1.59)	<.001
	Black	0.72 (0.68, 0.76)	<.001	0.78 (0.73, 0.83)	<.001	0.74 (0.66, 0.84)	<.001
	Another race	1.01 (0.86, 1.19)	0.90	1.01 (0.85, 1.21)	0.88	0.86 (0.64, 1.17)	0.35
	Unknown race	1.06 (0.90, 1.26)	0.47	1.07 (0.90, 1.28)	0.44	0.94 (0.69, 1.30)	0.72
Hispanic ethnicity	Non-Hispanic						
	Hispanic	0.88 (0.82, 0.94)	<.001	0.91 (0.84, 0.97)	<.001	0.97 (0.83, 1.12)	0.64
	Unknown	1.00 (0.93, 1.08)	0.96	0.99 (0.91, 1.07)	0.73	0.99 (0.84, 1.16)	0.87
Postmenopausal	Age <55 years						
	Age ≥ 55 years	0.96 (0.93, 1.00)	0.05	1.00 (0.96, 1.05)	0.86	1.04 (0.96, 1.14)	0.32
Stage	Unknown stage	0.19 (0.17, 0.20)	<.001	0.19 (0.18, 0.21)	<.001	0.29 (0.25, 0.33)	<.001
	Stage I	0.08 (0.07, 0.08)	<.001	0.08 (0.07, 0.08)	<.001	0.08 (0.07, 0.09)	<.001
	Stage II	0.18 (0.16, 0.19)	<.001	0.17 (0.16, 0.19)	<.001	0.16 (0.14, 0.18)	<.001
	Stage III	0.64 (0.60, 0.68)	<.001	0.63 (0.59, 0.67)	<.001	0.61 (0.55, 0.67)	<.001
	Stage IV	Reference					
Co-morbidities	0	0.90 (0.84, 0.97)	0.004	0.91 (0.84, 0.98)	0.01	0.92 (0.80, 1.07)	0.30
	1	0.90 (0.83, 0.97)	0.008	0.90 (0.83, 0.98)	0.01	0.88 (0.75, 1.04)	0.13
	2+	Reference					
Insurance	Private insurance	Reference					
	Medicaid	1.21 (1.13, 1.30)	<.001	1.22 (1.13, 1.32)	<.001	1.23 (1.05, 1.43)	<.001
	Medicare or other government	1.13 (1.09, 1.18)	<.001	1.12 (1.08, 1.16)	<.001	1.04 (0.97, 1.12)	0.29
	Uninsured	1.43 (1.30, 1.56)	<.001	1.44 (1.30, 1.58)	<.001	1.53 (1.22, 1.90)	<.001
	Unknown	1.21 (1.05, 1.40)	<.001	1.22 (1.05, 1.42)	<.001	1.02 (0.76, 1.35)	0.91
Median Income Quartiles	>= $74,063	Reference			<.001		
	$46,227-$57,856	1.09 (1.04, 1.15)	<.001	1.10 (1.04, 1.16)	<.001	1.06 (0.96, 1.18)	0.25
	$57,857-$74,062	1.05 (1.00, 1.10)	0.03	1.05 (1.00, 1.10)	0.04	1.08 (0.99, 1.18)	0.10
	< $46,277	1.04 (0.98, 1.11)	0.16	1.05 (0.98, 1.12)	0.17	1.04 (0.92, 1.18)	0.51
Percent No High School Degree Quartiles	>= 15.3%	Reference					
	5.0%-9.0%	0.98 (0.93, 1.03)	0.41	0.99 (0.93, 1.04)	0.62	0.98 (0.88, 1.10)	0.75

© 2025 Bodrutha Smith AJ et al. *JAMA Network Open*.

	9.1%-15.2%	0.99 (0.94, 1.04)	0.72	1.00 (0.95, 1.05)	0.99	1.05 (0.95, 1.17)	0.36
	< 5.0%	0.95 (0.89, 1.01)	0.08	0.94 (0.88, 1.01)	0.07	0.94 (0.83, 1.06)	0.32
Census Region	West	Reference					
	Midwest	1.00 (0.95, 1.05)	0.85	1.02 (0.96, 1.07)	0.57	0.99 (0.90, 1.10)	0.88
	North	0.97 (0.92, 1.02)	0.26	0.98 (0.93, 1.03)	0.42	0.94 (0.85, 1.04)	0.22
	South	1.09 (1.04, 1.14)	<.001	1.09 (1.04, 1.15)	0.0006	1.03 (0.94, 1.14)	0.50
Facility Type	Community	Reference					
	Academic	0.98 (0.95, 1.01)	0.25	0.98 (0.95, 1.02)	0.35	0.99 (0.93, 1.06)	0.78
Distance traveled for care		1.00 (1.00, 1.00)	<.001	1.00 (1.00, 1.00)	0.004	1.00 (1.00, 1.00)	<.001
Reported odds ratios are adjusted for all presented variables. Another race is a category from the National Cancer Database that includes all patients who do not identify as Asian, Black, or White.							

© 2025 Bodrutha Smith AJ et al. *JAMA Network Open*.
